# Traumatic aniridia with extensive episcleral pigmentation: a case report

**DOI:** 10.1186/s12886-022-02266-5

**Published:** 2022-02-08

**Authors:** Hsin-Yu Yang, Catherine Jui-Ling Liu

**Affiliations:** 1 Department of Ophthalmology, Taipei Veterans General Hospital, No. 201 Shipai Road, Sec. 2, Taipei, 11217 Taiwan; 2 Taipei Veterans General Hospital Yuanshan and Suao Branch, Yilan, Taiwan; 3 National Yang Ming Chiao Tung University School of Medicine, Taipei, Taiwan

**Keywords:** Traumatic aniridia, Phacoemulsification, Trabeculectomy, Transscleral cyclophotocoagulation, Endoscopic cyclophotocoagulation, glaucoma, Trabeculectomy, Contusion injury

## Abstract

**Background:**

Traumatic aniridia has been documented in eyes with a history of cataract extraction through a clear corneal wound. The proposed hypothesis is that the iris tissue was squeezed out from the corneal wound as it is a relative weak point. However, traumatic aniridia with extensive pigmentation of the episclera has never been reported.

**Case presentation:**

A patient, who has surgical histories of trabeculectomy and cataract surgery many years ago, presented with refractory high intraocular pressure (IOP), almost complete loss of the iris, and diffuse pigmentation of the episclera after he had suffered from a contusion injury. In addition to numerous pigment particles and cells in the anterior chamber and a well-centered intraocular lens, protruding uvea tissue with overlying conjunctiva adjacent to the site of trabeculectomy was noted. Gonioscopy showed absence of the iris with clear view of the ciliary body.

**Conclusions:**

The distinct presentation of this case indicates that the torn iris was displaced to the trapdoor instead of the clear cornea incision and was confined to the subconjunctival space. The scleral fistula serves as a less resistant point for releasing pressure compared to a healed corneal wound when the eye encounters a contusion injury. Further treatment options to lower IOP include repeated trabeculectomy, implantation of glaucoma drainage device, and endoscopic cyclophotocoagulation. Transscleral cyclophotocoagulation may be considered only after episcleral pigmentation has become less so as to avoid the risk of surface burn.

## Background

Contusion injury to an eyeball can cause a variety of ocular injuries, and the types and severity of injury depend on the mechanism and force on the spot: traumatic iritis, hyphema, cornea edema, lens dislocation, vitreous hemorrhage, commotio retinae, retinal detachment, scleral rupture, etc. [1] However, in eyes with a history of ocular surgery, the surgical wound usually plays as a weak point to release compression force. There are several reports of traumatic aniridia in eyes with a history of clear corneal phacoemulsification with a foldable IOL implantation (CCPI) [2–4], but reports on traumatic injury to eyes with previous CCPI and trabeculectomy are rare [5]. Herein, we report such a case which had an exceptional presentation.

## Case presentation

**Fig. 1:**
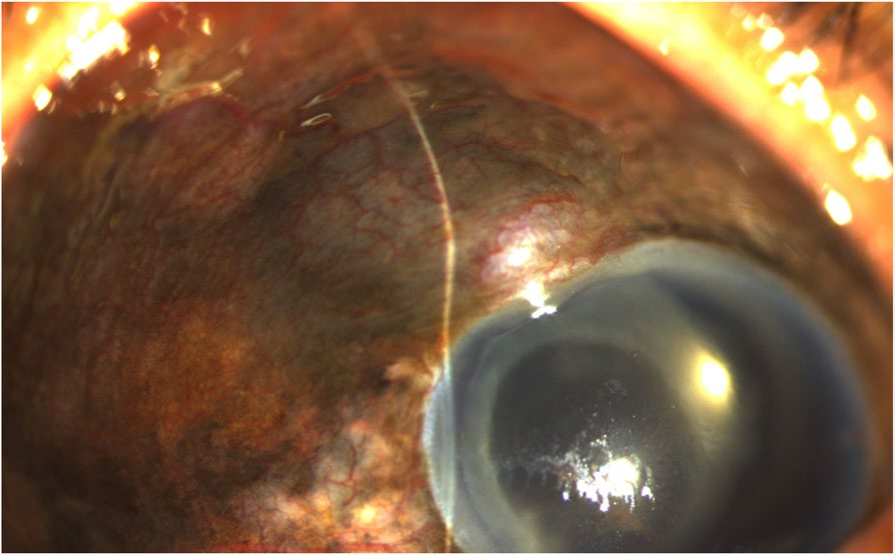
External eye photograph of the patient’s left eye (Zoom). Diffuse pigmentation of the episcleral and focal elevation of protruding uvea tissue at the superior-nasal episclera, which corresponds to the site of the trabeculectomy

A 55-year-old male presented with refractory high intraocular pressure (IOP) after hitting his left eyeball on a table corner in an accidental fall 3 days prior. The best corrected visual acuity was hand movement at 15 cm and IOP was 54 mmHg in the left eye. Slit Lamp examination showed an edematous cornea, diffuse black-brown discoloration of the episclera, a deep anterior chamber filled with numerous cells and pigment particles, complete loss of the iris except one strand of tissue in front of the capsular bag, and an intraocular lens (IOL) in situ without phacodonesis. Fundoscopic exam showed trace vitreous cells, attached retina with intact vasculature, and a pale disc. Tracing his history showed advanced glaucoma (Humphrey automated perimetry mean deviation, − 29.24 dB OD, − 21.99 dB OS), bilateral mitomycin C-augmented trabeculectomy 29 years prior, and CCPI in the left eye 8 years previous. The IOP had been controlled while on four topical glaucoma medications before this accident. An elevation in the superior-nasal aspect of the anterior sclera was noted, which showed protruding uvea tissue with overlying conjunctiva adjacent to the site of the trabeculectomy (Fig. 1).

**Fig. 2:**
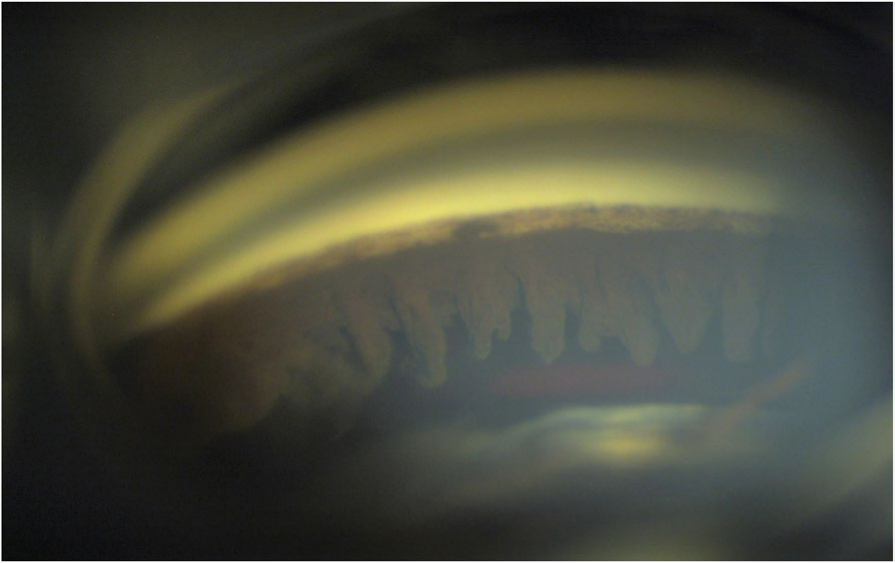
External eye photograph of the patient’s left eye. A photo taken two months later shows an intact capsular bag with the intraocular lens in situ. The iris is missing except one strand of remaining tissue

**Fig. 3:**
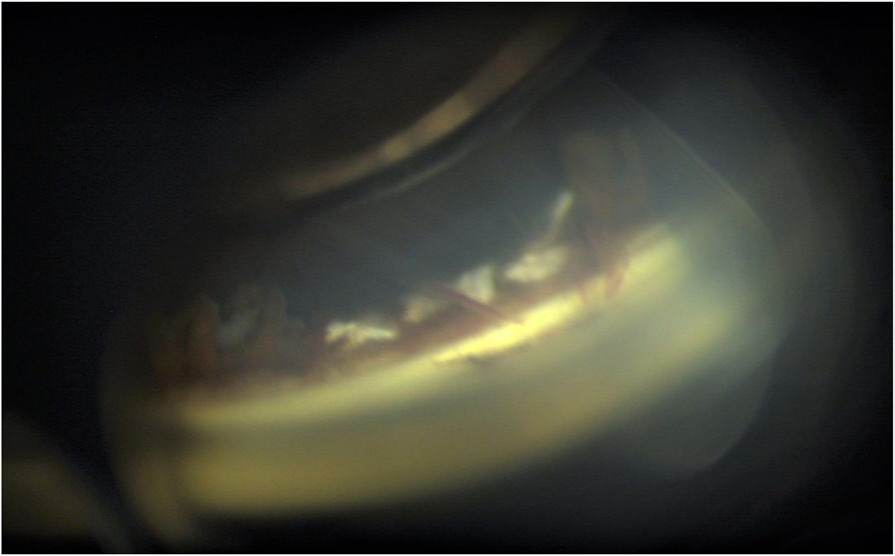
Gonioscopy of inferior angle. Two months later, gonioscopy revealed extensive loss of the iris, with ciliary processes shown

**Fig. 4:**
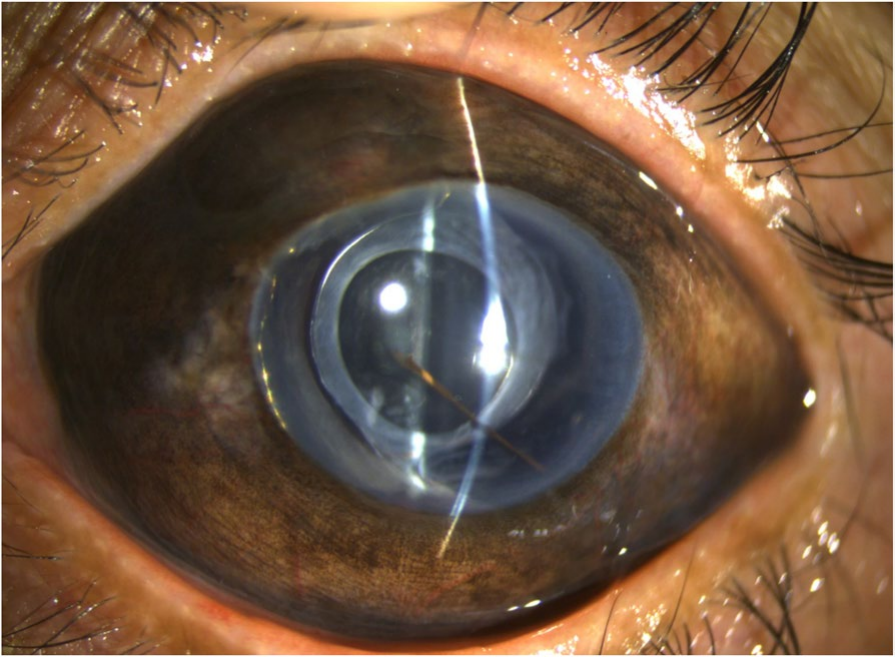
Gonioscopy of superior angle. Gonioscopy showed blood clots and pigment around the trapdoor

After weeks of topical steroid and antibiotic medications, intensive glaucoma treatment including oral acetazolamide, several times of intravenous infusions of mannitol and anterior chamber paracentesis, the anterior segment gradually cleared up (Fig. 2) and the IOP reduced to 28 mmHg. Gonioscopy revealed a missing iris over 360 degrees of the angle with clear view of the ciliary body (Figs. 3 and 4). Further treatment options including repeat trabeculectomy, glaucoma drainage device, endoscopic cyclophotocoagulation and transscleral cyclophotocoagulation were suggested for better IOP control. Due to poor optic nerve reserve, limited visual potential, and convenience of post-operative care, the patient chose to receive transscleral cyclophotocoagulation 5 months after trauma. In the latest follow up, which is 1 month after laser treatment, vision of the left eye improved to finger counting in front of 10 cm and IOP was 26 mmHg while under four topical antiglaucoma medications. Fundus exam revealed clear vitreous, attached retain and a pallor disc. However, the patient then lost to follow up.

## Discussion and conclusions

Expulsive iridodialysis, or traumatic aniridia is a clinical condition when the whole iris is torn from the ciliary body. It usually follows severe contusion injury of the eyeball. In a surgically naïve eye, this phenomenon often accompanies other injuries such as scleral rupture, lens dislocation, or commotio retinae [6, 7].

Regarding eyes with a history of ocular surgery, the wound from previous surgery is likely to become a weak point through where the transient high IOP is released. Several cases of traumatic aniridia and expulsive iridodialysis without IOL dislocation have been reported in eyes following CCPI [2–4, 8–12]. The researchers have speculated that the iris ejects via the wound upon the stroke, and then the wound closes spontaneously. Some have proposed that the elasticity of a fordable IOL can help absorb the striking force, thus prevents further damage to the zonule [3, 11].

We presented a unique case in which both trabeculectomy and CCPI had been performed many years before the accident. Since trabeculectomy aims at draining the aqueous humor from the anterior chamber to the subconjunctival space, it is not surprising that the torn iris was displaced to the trapdoor instead of the years-long corneal wound. The uveal tissue was largely incarcerated around the trapdoor and covered by the conjunctiva. The dense diffuse pigmentation of the episclera was stunning at first glance, indicating that a lot of pigment was flushed around the globe as the aqueous humor gushed from the anterior chamber.

The pigmentation on the episclera may become lighter in degree and limited in distribution as time goes by. Kaliperuma et al. reported a similar case who presented 4 months after trauma, and the subconjunctival pigmentation was mild and limited only to the superior fornix [5].

Anterior chamber paracentesis was conducted in an emergency situation to rapidly reduce IOP and decrease the amount of pigment particles and cells in the anterior chamber in an inflamed eye. When the eye quiets down and IOP remains uncontrolled with medications, further treatment options include repeat trabeculectomy at the superior-temporal aspect, glaucoma drainage device implantation, and endoscopic cyclophotocoagulation. Transscleral cyclophotocoagulation may cause surface burn when there is heavy pigmentation of the episclera, but may be performed when the pigmentation becomes lighter months later [13].

## Data Availability

The datasets used and/or analysed during the current study available from the corresponding author on reasonable request.

## Abbreviations

IOP: Intraocular pressure
CCPI: Clear corneal phacoemulsification with a foldable IOL implantation
IOL: Intraocular lens

## References

[1] Kiel J, Chen S (2001). Contusion injuries and their ocular effects. Clin Exp Optom.

[2] Ball J, Caesar R, Choudhuri D (2002). Mystery of the vanishing iris. J Cataract Refract Surg.

[3] Doro D, Deligianni V (2006). Ultrasound biomicroscopy in traumatic aniridia 2 years after phacoemulsification. J Cataract Refract Surg.

[4] Eom Y, Kang SY, Song JS, Kim HM (2013). Traumatic aniridia through opposite clear corneal incision in a pseudophakic eye. J Cataract Refract Surg.

[5] Kaliaperumal S, Troutbeck R, Iemsomboon W, Farinelli A (2014). Isolated traumatic aniridia after trabeculectomy in a pseudophakic eye. Indian J Ophthalmol.

[6] Gracner B, Pahor D (2001). Bilateral eye injury caused by a high-pressure water jet from a fire hose. Wien Klin Wochenschr.

[7] Loiudice P, Casini G. Post-traumatic iridodialysis, crystalline dislocation and vitreous haemorrhage: how to manage. BMJ Case Rep. 2014;2014:bcr2014205595. 10.1136/bcr-2014-205595 PMC4139545 25139923

[8] Zurutuza A, Andonegui J, Berástegui L (2011). Traumatic expulsive iridodialysis with vitreous prolapse. Int Ophthalmol.

[9] Walker NJ, Foster A, Apel AJ (2004). Traumatic expulsive iridodialysis after small-incision sutureless cataract surgery. J Cataract Refract Surg.

[10] Kahook MY, May MJ (2005). Traumatic total iridectomy after clear corneal cataract extraction. J Cataract Refract Surg.

[11] Muzaffar W, O'Duffy D (2006). Traumatic aniridia in a pseudophakic eye. J Cataract Refract Surg.

[12] Ruiz-Medrano J, Ávalos-Franco N, Gutierrez-Bonet R, Cifuentes-Canorea P, Gegundez-Fernandez JA, Diaz-Valle D (2017). Expulsive total iridodialysis through microincision phacoemulsification wound. J Fr Ophtalmol.

[13] Gaasterland DE (2009). Diode laser cyclophotocoagulation. Glaucoma Today.

